# A hybrid deep-learning-architecture for identifying cotton content in fabric materials

**DOI:** 10.1371/journal.pone.0346583

**Published:** 2026-05-05

**Authors:** Max Wiedemann, Christopher Mai, Luca Eisentraut, Ricardo Buettner

**Affiliations:** Chair of Hybrid Intelligence, Helmut-Schmidt-University/University of the Federal Armed Forces Hamburg, Hamburg, Germany; Mae Fah Luang University, THAILAND

## Abstract

Recycling plays a crucial role in achieving sustainable production. In particular, automating sorting processes holds great promise for enhancing both the efficiency and economic feasibility of the recycling industry. One challenge within this context is the classification of fabrics based on their cotton content. This task is relevant not only for recycling but also for the broader textile sector. Traditional methods often rely on manual labor, which is both time-consuming and labor-intensive, while advanced techniques like near-infrared spectrography, although effective, can be complex and expensive. We therefore propose a task-specific, deep-learning-based hybrid architecture approach for visually classifying fabrics based on their cotton content. The hybrid architecture leverages the strengths of DenseNet121 and Swin Transformer V2. The hybrid network is capable of capturing both local and global features, which enables it to detect differences in fiber types as well as quantify their presence within the fabric. To enhance its classification accuracy, we modified DenseNet121 with an adaptive feature pyramid network, which helps to consider features extracted at different levels, and a deformable convolution layer, focusing on structures in the fabric. Stratified 5-fold cross-validation was employed on a peer-reviewed dataset to assess the model’s performance and ensure its robustness. Compared to the state-of-the-art, we set a new benchmark for cross-validated visual approaches using standard camera imagery for cotton content classification with an average Root Mean Squared Error of 14.01%. We therefore prove the effectiveness of our architecture and its modifications. Our approach demonstrates the potential benefits of using deep learning methods for determining cotton content. These methods can help reduce manual effort, lower costs, and ultimately improve the economic situation of recycling companies.

## Introduction

To achieve the United Nations (UN) Sustainable Development Goals (SDGs), circular economy practices are a practical tool [1]. This is especially true for goal 12, which is about ensuring sustainable consumption and production patterns [1]. Substantially reducing the waste generation by 2030 is a central target of this goal, being measured purely by the national recycling rate and tons of material recycled [2]. Textile wastes are an issue rising simultaneously with the textile industry itself [3]. In textile production, cotton is one of the three most common materials, next to wool and polyester [4]. To reduce waste, the use of water, chemicals, and energy across the whole production chain, reusing and recycling cotton is an effective tool [5]. Additionally, composite reinforcements, bio-fuels, regenerated cellulose fibers, or other high-value products can be sourced from recycled cotton [3]. Correctly determining the material contents of the fabrics is a crucial step in the recycling process [6]. But doing this task manually is often time-consuming and labor-intensive [4]. The textiles labels are not reliable, as they are either not legible at the point of recycling or contain wrong information [6]. The latter is problematic not only for recycling purposes, but for end-consumers and the textile industry as a whole, as a fabric’s cotton content is an important measurement for its quality [7]. Because of these reasons, correctly determining the cotton content of fabrics is an important step in the manufacturing process, but the established methods for this are a complex process, involving toxicity and sample reconstruction [8]. A technique that emerged to avoid some of these issues is near-infrared (NIR) spectrography [6]. But the process can be costly and generates complex datasets which require sophisticated data analysis methods to interpret [9]. Visually examining the textiles is a less-researched field and can help to automate the process, reduce errors, and streamline the recycling process by making it more effective and efficient [4].

**Fig 1:**
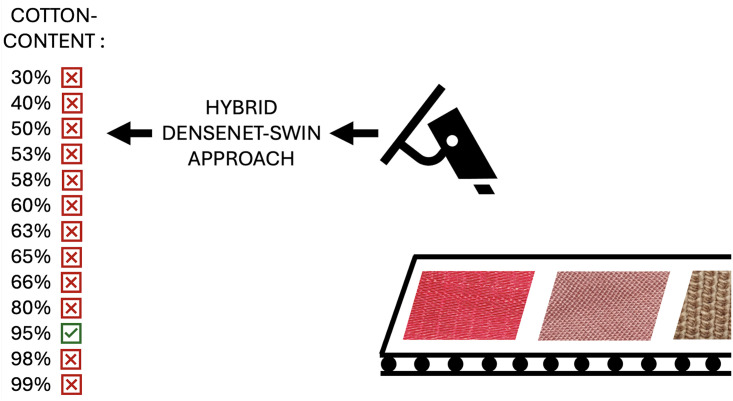
Visualization of a possible application of our approach to fabric classification.

NIR is an oftentimes utilized approach in research on classifying the cotton content of fabrics, especially on cotton-polyester blended fabrics [8,10,11], but approaches applying this method not on a specific fabric blend do exist as well [7]. Some research is also focused on cotton lint [12] or seed cotton [13] instead of cotton fabrics. These focuses are not applicable for classifying cotton contents. To our knowledge, Islam et al. [14] is the only study with a focus on visual methods and cotton fabric, and it currently has a benchmark of 7.56%. Their work does not use cross-validation. We address gaps in the literature of rare visual approaches and specific fabric blends by proposing a computer vision-based neural network (NN) to effectively recognize and classify the cotton content in different fabrics on a visual basis. Fig 1 is an example of how such a real-world application of a visual approach to fabric classification based on their cotton content could look.

In this work, we use a task-specific hybrid architecture approach, combining DenseNet121 with a Swin Transformer V2. We apply deformable convolution (DConv) layers and an adaptive feature pyramid network (AFPN) to enhance the feature representation and thereby the accuracy of our architecture. As the different fibers need to be inspected for differentiating between cotton and other materials, the frequency of those in the overall fabric tells us its cotton content. Because of this, small details and global features are both vital to correctly classify cotton content, which favors architectures that preserve fine texture details and capture spatial structure. The combination of local and global information is a task that the Swin Transformer V2 is especially good at [15]. We validated our hybrid approach with a stratified 5-fold cross-validation on a peer-reviewed dataset [4] and reached an average Root Mean Squared Error (RMSE) of 14.01%, setting a new benchmark for classifying cotton content.

The main contributions of this work are as follows:

1) By utilizing our task-specific architecture, we establish a reproducible benchmark performance for visual cotton content classification with an average RMSE of 14.01%.
2) We show that sophisticated methods for classifying cotton content can be visual-based and do not need to utilize NIR spectrography.

This work is structured as follows: Section Related Work lists and analyzes related works in the problem domain. Section Methodology displays the used methodology, including model architecture, training process, and the used dataset. Section Results shows the achieved results. These are critically discussed in Section Discussion, leading to a conclusion in Section Conclusion.

## Related work

### Classification of cotton content in fabrics

Classifying fabrics after their cotton content is a task useful in recycling as well as quality control [4]. But doing this task manually is time-consuming and cost-intensive [4]. A common approach to solve these problems for the task of classifying cotton content is NIR spectrography [6]. While this method is deemed effective, it is cost-intensive and creates complex datasets which are complicated to analyze [6]. A lot of related works try to analyze NIR spectrography data with regression models or deep learning (DL) methods. Some works in this field also focus on different tasks, such as classifying cotton-polyester blends [8]. Other research also does not focus on classifying cotton content in fabrics, but detecting faults in cotton lint, a waste product not usable for recycling [12]. Doing the task of classifying cotton content from fabrics visually is a way to automate the analog process and make it more time- and cost-effective [4]. Despite this, visual approaches to cotton content classification are rare.

### Deep learning in classifying cotton-content

The amount of literature on the subject of classifying cotton content using deep learning is not extensive. To our knowledge, we are the first to work with the CottonFabricImageBD dataset [4], which could be rooted in its novelty. But there is a larger amount of literature on classifying fabric contents with DL methods using other datasets. We also make a focus of the literature on near- and mid-infrared spectrography images. The literature on purely visual methods is limited.

#### NIR spectrography.

As mentioned, a large amount of research in the field of classifying cotton content deals with NIR spectrography. For example, dealing with cotton content but in their case of cotton-polyester blended fabrics, Xia et al. [8] use NIR spectrography with a CNN LSTM combination to classify the cotton content. The authors modified their CNN by replacing the last two convolutional layers with LSTM layers. With this approach, they lower the RMSE by 36.57% and 47.85% against different models in their testing set as well as their validation set, and therefore achieve a higher accuracy in classifying cotton contents. They achieved an RMSE on the validation set of 0.65% and 0.75% on the testing set. Also using NIR image spectrography, Sun et al. [11] applied different preprocessing methods such as multiplicative scatter correction, first derivative or second derivative, and Monte Carlo uninformative variables elimination, successive projections algorithm, and genetic algorithm to various partial least squares regression models. Their optimal model used preprocessing of 2 Der-Smooth-MSC and a variables selection method of MCUVE-SPA-PLS, achieving an *r*^2^ of 0.988% and a root mean squared error of prediction (RMSEP) of 2.1%. By this, they showed that cotton content in cotton and polyester textile blends could be determined by NIR spectroscopy. Paz et al. [10] also focused on NIR as well as mid-infrared spectrography on cotton polyester blends and applied different models, such as principal component analysis, partial least squares discriminant analysis, and partial least squares regression, also proving the latter is a usable technique for cotton-content estimation. Their PLS model performed best and achieved a calibration error of 3.3% and RMSEP of 3.6% for NIR spectrography data. As well, with a focus on NIR spectrography data, Tao [7] quantitatively analyzes the data. They compare a deeper model with a model with attention mechanisms across eight different datasets, with the label and noise balancing methods applied. The CNN performs generally better than the traditional neural network in their scenarios. Li et al. [13] proposed a NIR spectrography-based method to analyze impurity content in machine-picked seed cotton and applied a CNN utilizing different activation functions for data analysis. They achieved an *r*^2^ of 0.9063 and an RMSE of 0.0546.

#### Visual approaches.

Other research focuses on visual approaches rather than NIR spectrography; relevant examples are outlined in the following. With the use of a multi-class SVM, Li et al. [12] wanted to classify foreign fibers in cotton lint. Trying to classify six different classes, namely hair, black plastic film, red cloth, hemp rope, red polypropylene, and black feather. Comparing different SVM approaches separately, such as one-against-all decision-tree based MSVM, one-against-one voting-based MSVM, and one-against-one directed acyclic graph MSVM, they reach an accuracy of 93.57% with a one-against-one voting-based approach and 92.34% with a directed acyclic graph approach. Only these two approaches reach their accuracy requirements, with the one-against-one directed acyclic graph MSVM being the fastest in classification speed of all three approaches. Closer related to our work is the study of Islam et al. [14] who did a cotton-content prediction from fabric images, using a CNN with transfer learning and reached an RSME of 7.56%. They did not use a cross-validation approach in combination with a photometric stereo sensor dataset. We address these limitations by using a stratified 5-fold cross-validation, as well as a peer-reviewed dataset containing images captured with a normal camera. We also do not focus on prediction, but on the classification of the cotton contents. Table 1 provides a short overview of the described related work.

**Table 1:** Overview of related work for fabric classification.

Author	Year	Cross-Validated Approach	No. of Classes	Visual Approach	Cotton Fabric	Method	Results
Islam et al. [14]	2023	×	34	✓ (Photometric Stereo Sensor)	✓	VGG16 with transfer learning	RMSE: 7.56%
Li et al. [12]	2010	✓	6	✓	×	Multi-class SVM	Accuracy: 93.57% / 92.34%
Li et al. [13]	2024	×	N/A	✓	×	CNN with different activation functions	*r*^2^: 0.9063 RMSE: 0.0546
Paz et al. [10]	2024	✓	4	×	✓	Partial least squares regression	RMSEP 3.6%
Sun et al. [11]	2016	✓	N/A	×	✓	Partial least squares regression models	*r*^2^: 0.988% RMSEP: 2.1%
Tao [7]	2023	✓	N/A	×	✓	CNN	The comprehensive evaluation demonstrates that CNNs consistently outperform linear methods (RMSE 0.114 on modal cotton)
Xia et al. [8]	2024	✓	N/A	×	✓	CNN-LSTM combination	RMSE: 0.65%
**This study**	**2025**	✓	**13**	✓	✓	**Hybrid Architecture with DenseNet121 + Swin Transformer V2**	**RMSE: 14.01%**

#### Research gap.

The listed related works show a very extensive amount of literature dealing with NIR spectrography-based approaches. Additionally, oftentimes, regression models are used for the cotton content classification rather than DL methods. In the cases where literature does not apply NIR-spectrography, it oftentimes does not deal with fabrics but cotton lint or seed cotton instead. As we aim to classify the cotton content of fabrics, those approaches are not relevant for the scope of our work. Additionally, two related works do not use cross-validation approaches and do not use peer-reviewed datasets, limiting their reliability and reproducibility. To not be dependent on the costly and complex NIR spectrography [9], we address this research gap of a limited number of studies utilizing visual approaches. By applying a task-specific hybrid architecture approach, we also apply a DL method to this field rather than a simpler regression model. We chose to combine DenseNet121 with a Swin Transformer V2 to have global as well as local feature representation. We modified DenseNet121 to enhance feature representation and overall accuracy of our approach, the modifications used are outlined in methodology section.

## Methodology

**Fig 2:**
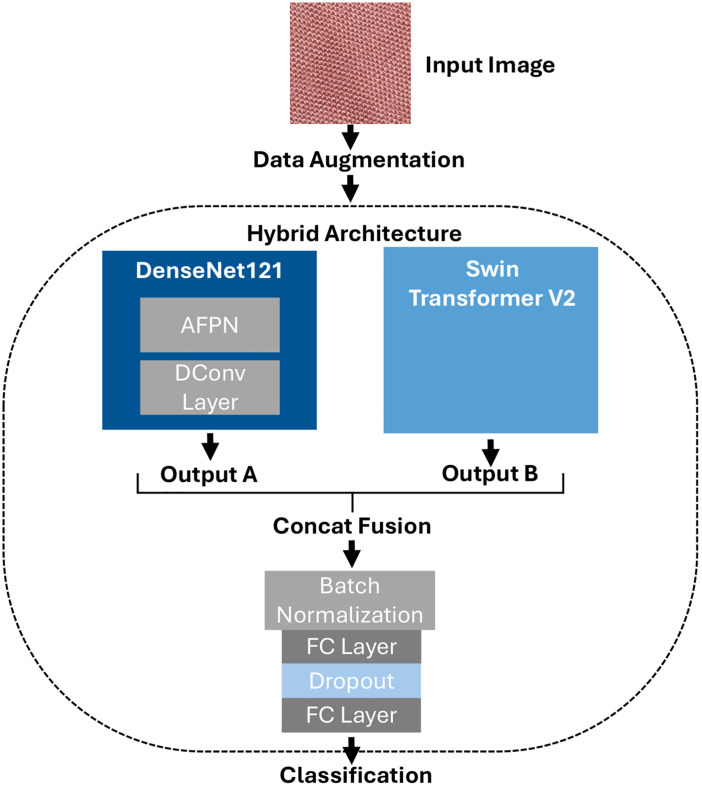
Schematic visualization of the hybrid architecture.

Our hybrid architecture combines DenseNet121, modified with a DConv layer and an AFPN, with Swin Transformer V2. The used modules and methods, as well as the overall architecture of our model, are discussed in this section. A schematic visualization of this approach is represented in Fig 2.

### Model architecture

#### Hybrid architecture.

**Fig 3:**
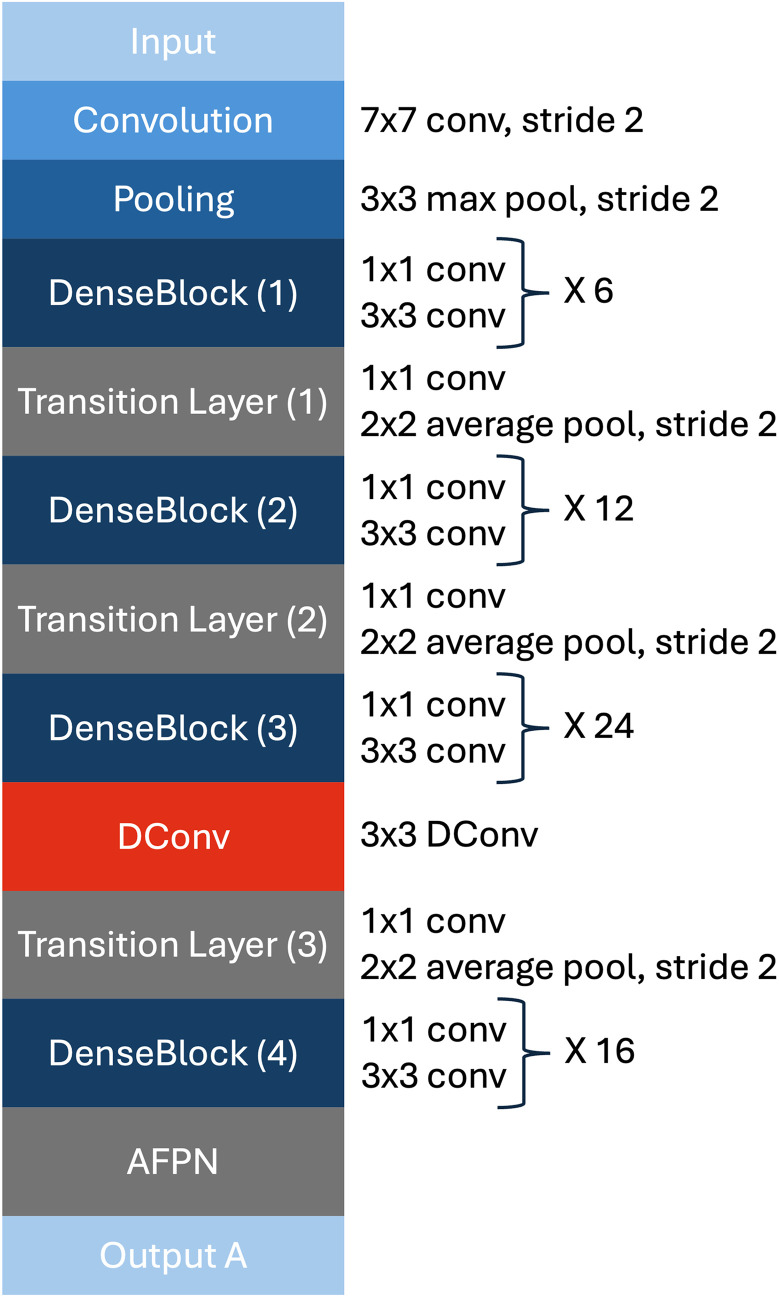
Visualization of the DenseNet121 architecture, in which an AFPN and a DConv layer is integrated between DenseNet blocks three and four.

We used transfer learning in comparison to training an entire model from scratch. As mentioned, the base models used in this study are DenseNet121 and a Swin Transformer V2. DenseNet121 was chosen over other backbones because it is particularly well suited for representing dense features and thus, for example, subtle tissue structures across all layers. This is not necessarily the case to the same extent with other backbones, such as those that work heavily with residual connections. The Swin Transformer V2 was selected because its shifting window attention makes it particularly well suited for processing spatially repetitive features, which is less the case with transformers that use token mixing [23]. We modified DenseNet121 with DConv layers in between DenseBlocks three and four (see Fig 3), as well as an AFPN. Each of the two architectures independently extracts relevant features from the input images. The outputs of both architectures are then combined. The initial two-dimensional results of both models are simplified to a one-dimensional feature map by adaptive averaging and flattening. These 1D vectors are then processed by batch normalization. Mathematically, batch normalizations work applying the Eq. (1) and (2) [16]. Eq. (1) displays the normalization step, while Eq. (2) displays the following step of scaling and shifting. Here *x*_*i*_ are activations, *B* is the batch size, and $\sigma_{B}^{2}$ is the batch variance, while ϵ is a constant added for numerical stability. γ and β are to be learned parameters, and BN is the batch-normalization transform. Finally, *y*_*i*_ are the scaled and shifted values [16]:


$${\hat{x}}_{i}\leftarrow \frac{x_{i}-\mu_{B}}{\sqrt{\sigma_{B}^{2}+\epsilon }}$$ (1)

$$y_{i}\leftarrow \gamma {\hat{x}}_{i}+\beta \equiv {\text{BN}}_{\gamma ,\beta }(x_{i})$$ (2)

These one-dimensional outputs of both are then fused via the concatenation function and batch normalization is applied again. This second normalization re-aligns the feature statistics after fusion, preventing scale discrepancies between the CNN and Transformer representations. After the fusion, a new vector ${𝐂}^{n_{A}+n_{B}}$ is created where $𝐀\in {\mathbb{R}}^{n_{A}}$ and $𝐁\in {\mathbb{R}}^{n_{B}}$ are the two output vectors of DenseNet121 and the Swin Transformer V2, *n* is the length of the respective vector. The Swin Transformer V2 combines different areas of the input image to recognize how these are connected. Thereby, local information is combined with global information [15]. This helps in our case to detect different fibers and connect this information to the overall structure of the fabric, enabling the network to better detect the cotton content of the respective fabric. Through the use of two FC layers after the concat fusion, we aimed to connect the extracted differences in order to enhance classification accuracy. This is needed especially for classifying cotton contents with small percentage differences and by that enhance the model’s classification capacity, a task for which using more FC layers is considered useful [17]. Two fully connected layers were selected empirically, as preliminary tests had shown that a single FC layer resulted in lower validation performance. An additional FC layer was not used due to the increasing number of parameters and complexity. The number of neurons in the first FC is determined by hyperparameter tuning, the number of neurons for the second FC is 13, the number of classes.

#### DenseNet121.

We chose DenseNet121 as one part of our hybrid architecture. The basic idea of Densenet121 is based on the fact that CNNs are oftentimes more accurate and their training is more efficient, when the connections between the layers close to the input and the output are shorter [18]. Therefore, DenseNet121 connects each layer to every other layer in a feed-forward fashion, so the outputs of all preceding layers are used as inputs for a layer and its outputs are inputs for all following ones. All extracted features are fused by a composite function of the three consecutive operations of a batch normalization, a ReLu function, and a 3 × 3 convolution. Before this 3 × 3 convolution, a 1 × 1 convolution is applied as a bottleneck layer to improve computational efficiency and reduce the number of feature maps [18]. This is how the so-called dense blocks work, which are connected through transition layers that change feature map sizes through convolution and pooling [18]. A DConv layer was inserted between Dense Blocks 3 and 4 (as can be seen in Fig 3) to address irregularities in the fiber structure, allowing the model to capture subtle differences. keeps the same number of input and output channels (1024) as the preceding block output. The deformable convolution uses: kernel size = 3, stride = 1, padding = 1 and dilation = 1. This modification does not change the spatial or channel dimensions. The AFPN module receives the outputs of the transition layers (1–3) of DenseNet121 (128, 256, and 512 channels and 56x56, 28x28, 14x14 resolutions) and constructs a three-level feature pyramid. First, each level is projected to 256 channels via 1×1 and 3×3 convolutions with Batch Normalization. Top-down fusion then merges the higher- and lower-level features. In the adaptive variant, learnable Softmax-normalized weights *w*_*i*_ modulate each level, enabling the model to emphasize the most relevant scale. The three weighted feature maps (in total 768 channels) are concatenated and reduced to 512 channels via a 3×3 convolution. This forms the final, multi-scale feature map. The architecture of the modified DenseNet121 is displayed in Fig 3. Differently to ResNet, DenseNet121 does not sum features before passing them into a layer, but concatenates them. Through this, the $\ell^{\text{th}}$ layer has ℓ inputs. Further, DenseNet layers are only adding a small set of feature maps to the combined knowledge of the network, as they are very narrow with a small number of filters per layer. This encourages feature reuse, strengthens feature propagation, and reduces the number of parameters [18]. Additionally, DenseNet121 has an implicit supervision as each layer has direct access to the original input signal as well as the gradients from the loss function, improving information flow through the network and helping with training, especially in deeper architectures [18].

#### Deformable convolution layer.

**Fig 4:**
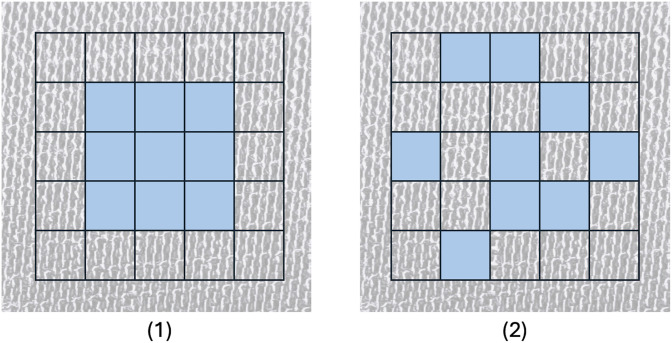
Visualization of deformable convolution with the example of a 3×3 convolution filter. (1) normal convolution, (2) deformable convolution.

DConv layers can adapt to specific structures or objects in the data, making them useful for our task of detecting cotton content, as the differences here are based on the structures of the fabrics. They add learnable offsets to the sampling positions within the filter, making them no longer fixed (see Fig 4) [19]. With these offsets, which are calculated through additional convolution layers, the network can adapt and detect specific objects or structures in the data [20]. This is especially interesting when detecting cotton content, as different structures of fibers are a differentiating factor. They work by applying Eq. (3), where *p*_0_ is the processing pixel, $\Delta p_{n}$ is the deformable convolutions offset, and *R* is sampling the regular grid of convolution:


$$y(p_{0})=\sum_{p_{n}\in R}w(p_{n})\cdot x(p_{0}+p_{n}+\Delta p_{n})$$ (3)

#### Adaptive feature pyramid network.

**Fig 5:**
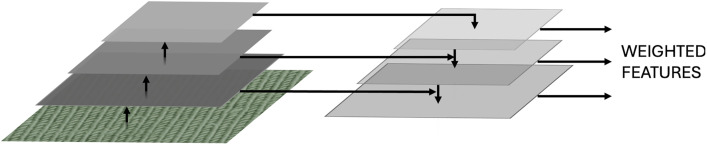
Illustration of an AFPN.

Feature Pyramid Networks (FPNs) combine features from different levels of the resolution through addition, making the network better at considering features from global as well as detailed levels [21]. AFPNs are, like FPNs used to represent extracted features at multiple levels of the resolution, where higher feature maps are merged with with lower levels, being different in assigning adaptive weights to each of these feature maps before merging, to decide which features are included how much in the final feature map [22]. AFPNs are visualized in Fig 5. They help make the network consider features extracted at different levels and therefore focus more on overall structures.

#### Swin transformer V2.

**Fig 6:**
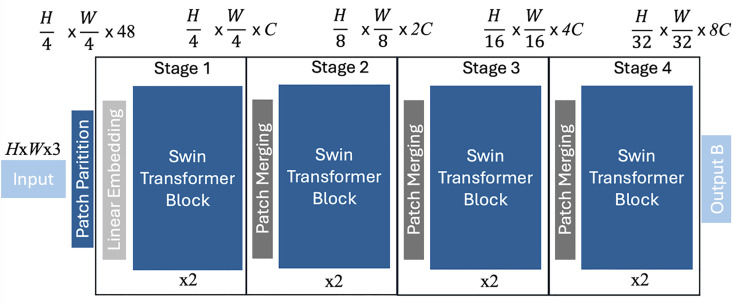
Visualization of the Swin Transformer architecture.

As it is one part of our hybrid architecture, the Swin Transformer V2 is an essential part of our approach. The Swin Transformer uses hierarchical feature maps by merging image patches [23]. With this technique, global features are combined with local features, representing information from both levels [15]. As Swin stands for Shifted WINdows, the shift is a key design element of the Swin Transformer [23]. Here, the window partition between two consecutive layers shifts, making the windows bridge edges of the ones from the preceding layer, which enhances the modeling power of the Swin Transformer significantly, as well as making it more efficient regarding latency [23]. The Swin Transformer down-samples the resolution by 2x and thereby reduces the amount of tokens equally in each layer as the network gets deeper to represent features from each layer hierarchically [23]. Additionally, the shifted window approach of the Swin Transformer has a much lower latency than other sliding window approaches and linear computational complexity to image size, making it suitable for a variety of tasks [23]. How the Swin Transformer functions is visualized in Fig 6. In this figure, displayed Swin Transformer block replaces the multi-head self-attention (MSA) in a transformer block with a module based on shifted windows, which is followed by a 2-layer Multi-Layer Perceptron (MLP) with Gaussian Error Linear Unit (GELU) non-linearity in between and a LayerNorm layer applied before, as well as a residual connection applied after each MSA and MLP module [23]. The Swin Transformer uses a patch size of 4 × 4, because of which the feature dimension of each patch is 4 × 4 × 3 = 48, and on this raw-valued feature, a linear embedding layer, denoted as *C*, is applied to project it to an arbitrary dimension [23]. The second version (V2) of the Swin transformer enhanced training stability and overall enhanced accuracy, due to a new normalization configuration which produces milder activation values as well as scaling up the model capacity [15]. The Swin Transformer is generally well-suited for classification tasks [23]. In our use case, it is additionally helpful to enable the network to detect local features such as different fabric structures, as well as the number of those fibers, a global feature.

### Process of training

**Fig 7:**
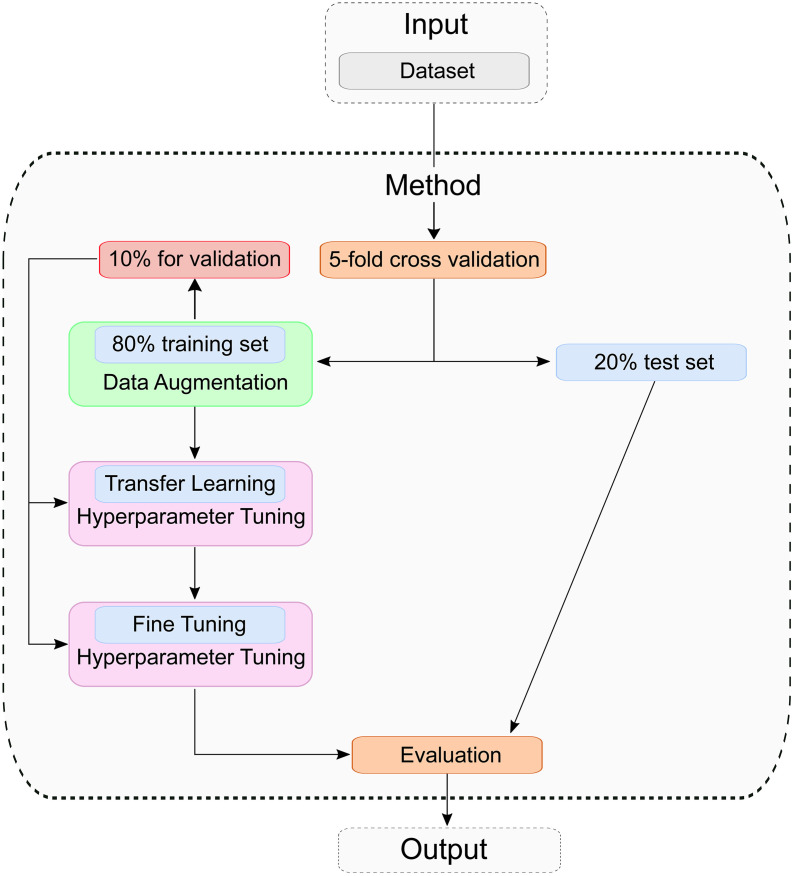
Training and Evaluation approach: The dataset is split into a training set and a test set. Data augmentation is applied to the training set, followed by model training using transfer learning and fine-tuning. Finally, the resulting model is evaluated on the test set.

The entire training process is shown in Fig 7. Before model training, a stratified 5-fold cross-validation was applied using the scikit-learn library [24] to divide the dataset into five equally sized subsets. This method was chosen to ensure that each fold maintains the original class distribution, unlike standard k-fold cross-validation, which may produce biased results due to class imbalance. In each iteration, four folds (80%) are used for training, and one fold (20%) is held out for testing. This process is repeated five times, rotating the test fold each time to ensure robust performance evaluation. Within the training set of each fold, 10% is further reserved for validation and hyperparameter tuning. All images were resized from 900×1200–256×256 pixels using bilinear interpolation with anti-aliasing to avoid aliasing artifacts, and subsequently normalized using the ImageNet mean and standard deviation for the three RGB channels. This input size ensures artifact-free patch partitioning in Swin Transformer V2 and provides slightly higher spatial detail compared to 224×224, allowing fine fabric textures to be better preserved. To reduce overfitting and improve generalization, the training data is augmented using RandomRotation(±10°) and RandomResizedCrop(scale=(0.9, 1.0)) as well as RandomAffine(translate=(0.1, 0.1)). These transformations account for variability in image scale and position, making the model more robust to input variations.

The techniques used were chosen conservatively in order to preserve the texture-relevant characteristics of the fabric, which are crucial for classifying the cotton content. Slight geometric transformations are used. In order not to distort the structure of the fabric too much and not to reduce the model performance, the parameter interval was limited to smaller values, these smaller intervals are also based on previous work. All transformations are applied on-the-fly during training. The chosen augmentations aimed to maintain texture features important for identifying cotton content. These augmentations do not change the weave structure, but rather simulate realistic variations in recording conditions. This preserves the fiber orientation, weave pattern and local texture. These data augmentation techniques are only applied to the training data. The validation and test data are not augmented. Each model is trained for a maximum of 100 epochs with a batch size of 16. The training consists of two phases: transfer learning and fine-tuning. To ensure stable optimization and comparability, the batch size was fixed to 16 during transfer learning, since only the classification head was trained. During fine-tuning, we explored batch sizes of 8, 16, and 32 to assess their influence on convergence and generalization when all layers were unfrozen. We selected a batch size of 16, as smaller batches can lead to unstable gradients, while a batch size of 32 or more increases memory consumption. During transfer learning, the pretrained feature extraction layers (based on ImageNet weights [25]) are frozen, while the fully connected layers remain trainable. In the fine-tuning phase, the entire model is updated. For both training phases, we used Bayesian optimization based on the Tree-Structured Parzen Estimator (TPE, Optuna) for sequential hyperparameter selection. The following hyperparameters are used: dropout, learning rate, weight decay fully connected layer units, batch size, and the optimizer. The corresponding interval can be seen in Table 2. For each fold, 20 trials are conducted and the configuration that yields the lowest validation loss is selected. At each optimization step, optuna saves the lowest validation loss. At the end of the hyperparameter optimization, all 20 validation losses are compared, and the lowest one is output with the corresponding parameter combination. Each trial is allowed to run up to 100 epochs. To avoid overfitting and reduce computational overhead, early stopping is implemented, terminating training if the validation loss does not decrease over ten consecutive epochs. Early stopping does not end the entire optimization process, but only the training process of the respective trial. During training, a constant learning rate was used and no learning rate schedule. After identifying the optimal hyperparameters, a final transfer learning model is trained using the same configuration as during optimization. The model with the lowest validation loss is saved and used as the base for the subsequent fine-tuning phase. During fine-tuning, the hyperparameters (see Table 2) are optimized again. To accomplish this, the entire model is unfrozen to allow for additional domain adaptation. Again, the best-performing model based on the lowest validation loss is stored. CrossEntropy is used exclusively as the loss function for the entire training process. Although additional distance metrics such as RMSE and Mean Absolute Error (MAE) are used for evaluation in this study, no other loss function is used. These metrics are calculated exclusively after training based on the predicted classes. After training, the final model is evaluated on the 20% test set using the following performance indicators such as accuracy, balanced accuracy, RMSE, MAE, true positive rate (sensitivity or recall), true negative rate (Specificity), positive predictive value (precision), negative predictive value, Cohen’s Kappa, and F1-score. The model outputs logits, and class predictions are obtained via argmax. The results are reported based on the model’s native outputs, without a post-hoc calibration. All these metrics are computed for each fold, stored, and finally averaged across all five folds to provide a comprehensive overview of model performance.

**Table 2:** Overview of the hyperparameters used for hyperparameter tuning. TL = Transfer learning; FT = Fine-Tuning.

Hyperparameter	Minimum Value	Maximum Value	Step	TL or FT
Dropout	0.1	0.5	0.05	TL, FT
Learning rate (TL)	10^−4^	10^−2^	calculated logarithmically	TL
Learning rate (FT)	10^−6^	10^−4^	calculated logarithmically	FT
Weight decay	10^−5^	10^−3^	calculated logarithmically	TL, FT
1. FC layer units	128	1024	128	TL, FT
Batch size	–	–	8, 16 or 32	FT
Optimizer	–	–	AdamW, SGD	TL

### Evaluation metrics

For the evaluation of our model, we applied the metrics (Balanced) Accuracy, true positive rate (sensitivity or recall), Positive predictive value (precision), true negative rate (Specificity), negative predictive value, and Cohen’s Kappa, as well as the F1-Score. These metrics relate to the multiclass case. Weighted metrics provide a more realistic assessment of performance when the dataset is unbalanced. When the dataset is balanced, the result corresponds to that of the macro metric. In addition to these classification metrics, we also report RMSE and MAE. While many related studies treat cotton-content prediction as a regression task using distance-based metrics such as RMSE, the present dataset defines fixed percentage levels as discrete classes, reflecting industrial practice where cotton textiles are produced with standardized compositions. A classification formulation is therefore appropriate, yet the numerical distances between classes remain relevant. Consequently, RMSE and MAE serve as complementary metrics that quantify how far the predicted percentage class deviates from the true value on this ordered scale, with RMSE emphasizing larger deviations and MAE capturing the average absolute error. The overall effectiveness of a model is displayed through the accuracy [26]. The balanced accuracy is used to avoid inflated accuracy scores with imbalanced datasets [24]. The accuracy is calculated by Eq. (4), the balanced accuracy by Eq. (5). Balanced accuracy is calculated as the arithmetic mean of the true positive rate TPR_*i*_ for each class i∈{1,..,N} [24]:


$$\text{Accuracy}=\frac{TP+TN}{TP+TN+FP+FN}$$ (4)

$$\text{Balanced\textasciitilde{}Accuracy}=\frac{1}{N}\sum_{i=1}^{N}\frac{TP_{i}}{TP_{i}+FN_{i}}$$ (5)

RMSE quantifies how far predicted values deviate from the true values on average. It is defined as the square root of the mean squared error, where the squared differences between predictions and ground-truth values across all samples are averaged [27]. The MAE captures how much predictions differ from the true values on average, based on absolute deviations [27]. In our case, we use the percentage values of the cotton content associated with each class and compute the deviation between predicted and true percentages. This provides a reliable measure of the magnitude of misclassification on the underlying ordinal scale, as it explicitly accounts for the numerical distances between classes. The RMSE is given by:


$$\text{RMSE}=\sqrt{\frac{1}{N}\sum_{i=1}^{N}(y_{i}-{\hat{y}}_{i}{)}^{2}}$$ (6)

$$\text{MAE}=\frac{1}{N}\sum_{i=1}^{N}|y_{i}-{\hat{y}}_{i}|$$ (7)

The true positive rate (TPR) shows how accurately the model can correctly classify the positive class. When it is maximized, the likelihood of correctly identifying true members of the positive class is increased [28]. For multiclass tasks, the TPR is computed as a weighted average, with each class weighted according to its number of samples [29]. The equation is shown in 8. The positive predictive value (PPV) indicates the proportion of positive predictions that correctly match true positive instances and, by that, displays the prediction accuracy for the positive class [28]. Both are calculated using the Eq. (8) and (9), where *n*_*i*_ represents the number of samples per class and *S* denotes the total number of samples:


$$\text{TPR}=\sum_{i=1}^{N}\frac{n_{i}}{S}\cdot \frac{TP_{i}}{TP_{i}+FN_{i}}$$ (8)

$$\text{PPV}=\sum_{i=1}^{N}\frac{n_{i}}{S}\cdot \frac{TP_{i}}{TP_{i}+FP_{i}}$$ (9)

The true negative rate (TNR) is analogous to the TPR, the measure of how effectively the negative classes are classified as such [26]. In the multiclass case, TNR is computed per class using a one-vs-rest strategy and aggregated using negative-support weights *TN*_*i*_ + *FP*_*i*_ per class. Similar to its positive equivalent, the negative predictive value (NPV) is the ratio of correctly classified negative samples to all samples classified as negative [30], in the multiclass case, NPV is computed per class using a one-vs-rest strategy and aggregated with *TN*_*i*_ + *FN*_*i*_ per class. Analogue to their positive counterparts, these are displayed below in the Eq. (10) and (11):


$$\text{TNR}=\frac{\sum_{i=1}^{N}(TN_{i}+FP_{i})\;\cdot \frac{TN_{i}}{TN_{i}+FP_{i}}}{\sum_{i=1}^{N}TN_{i}+FP_{i}}$$ (10)

$$\text{NPV}=\frac{\sum_{i=1}^{N}(TN_{i}+FN_{i})\;\cdot \frac{TN_{i}}{TN_{i}+FN_{i}}}{\sum_{i=1}^{N}TN_{i}+FN_{i}}$$ (11)

Cohen’s kappa describes the reliability of a model by measuring the agreement between two judgments. It ranges from −1–1, where a Cohen’s kappa of −1 indicates complete disagreement, and a Cohen’s kappa of 1 signifies perfect agreement. Cohen’s kappa is calculated using the Eq. (12), where *p*_0_ is the number of times the predicted value matches the actual value and *p*_*c*_ is the proportion of matches that would be expected by coincidence [31]:


$$Kappa=\frac{p_{0}-p_{c}}{1-p_{c}}$$ (12)

The F1-score is computed as the harmonic mean of precision and recall, ranging from 0 to 1, where 0 indicates that all positive samples are misclassified and 1 represents perfect classification [32]. This equation for multiclass [33] is visualized below as Eq. (13):


$$\text{F1-Score}=\sum_{i=1}^{N}\frac{n_{i}}{S}\;2\cdot \frac{PPV_{i}\cdot TPR_{i}}{PPV_{i}+TPR_{i}}$$ (13)

### Dataset

**Fig 8:**
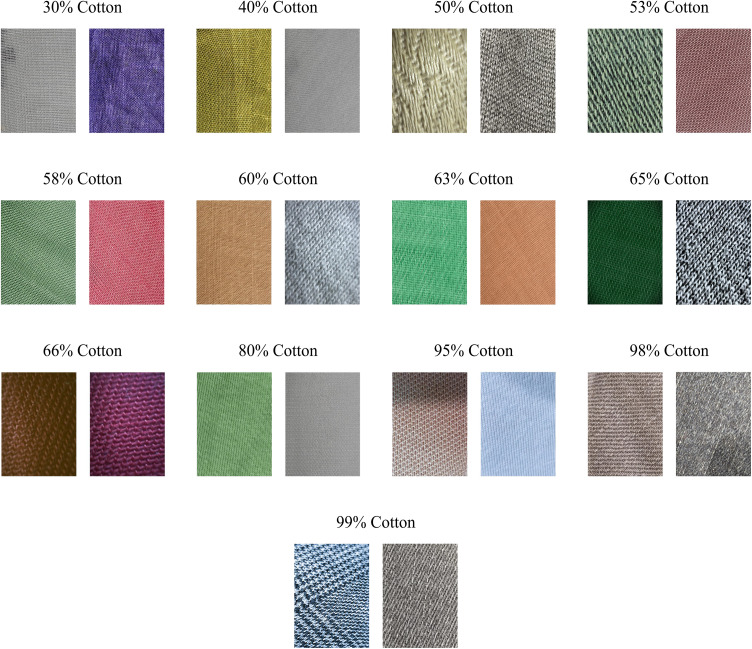
Example images from the dataset for each class. Shown are two images per cotton content class (13 classes in total) [4].

To evaluate our novel hybrid approach, we use the CottonFabricImageBD dataset [4]. In total, 1,300 original fabric images are provided, with a typical image resolution of about 900 × 1200 pixels. The images are labeled in thirteen classes representing the following cotton contents: 30%, 40%, 50%, 53%, 58%, 60%, 63%, 65%, 66%, 80%, 95%, 98%, and 99%. Each class contains 100 images. Fig 8 shows examples of images at different cotton content levels to compare the images visually. The images in each class vary in colors and patterns. The authors sourced the fabrics from different sources in Bangladesh, including fabric stores and clothing manufacturers. Images of the fabrics were captured with a 48 MP (f/2.0, (wide), 1/2.0″, 0.8 μm, PDAF) camera. The images were taken under consistently good lighting conditions using automatic exposure. They therefore represent a semi-controlled environment, as would be expected in real production results. Under the supervision of two textile engineering experts, they used a thread-counting machine to assess the cotton percentage of each fabric. In this process, the authors utilized the counting grid of the thread-counting machine and calculated the cotton content using the traditional thread count formula, a common practice in the textile industry. The measurement accuracy or error margin of the thread-counting machine is not mentioned. Through this process, 100 images in each class were sourced, resulting in a total of 1,300 images.

### Setup

For training and testing the architecture, a NVIDIA L40S GPU with 48 GB of memory, with PyTorch 2.5.0 is used. Furthermore, Python version 3.11.7 and CUDA version 12.4.1 were used. The architecture was trained with the best optimizer found in the hyperparameter tuning for a maximum of 100 epochs in various phases, such as transfer learning and fine-tuning. To identify the optimal parameters for transfer learning and fine-tuning, hyperparameter tuning was performed in 20 trials using Optuna (version 4.2.1). To avoid overfitting and save computation time, the callback function *early stopping* with a *delta* = 0.001 was used, which stops the training after 10 consecutive epochs in which the validation loss has not decreased. Scikit-learn (version 1.5.2) was utilized for stratified cross-validation and the computation of performance indicators. Throughout the entire training and validation process, the images were converted to a resolution of 256×256 pixels.

## Results

The performance metrics are presented in Table 3. The F1-score, TNR, TPR, PPV, and NPV were computed using weighted averaging. As shown in the table, the presented architecture achieves an RMSE of 14.01%, indicating robust estimation of cotton content overall. Table 4 also shows the results per class. The total training time was 2 hours and 22 minutes, and the model achieved an average latency of 4.09 ms per image with a computational load of around 43.7 GFLOPs. The total number of parameters varies across the 5 folds due to the changing number of units in the first FC layer and is as follows: (fold 1, 896 units): 110,954,416; (fold 2, 640 units): 110,422,960; (fold 3, 1024 units): 111,220,144; (fold 4, 1024 units): 111,220,144; (fold 5, 896 units): 110,954,416. By evaluating our architecture using stratified 5-fold cross-validation, we achieved an accuracy and balanced accuracy of 50.23%. The TPR indicates that, across all five folds an average of 50.23% of the images validation set, which were classified as belonging to a class, are correctly identified as having their respective cotton content. The TNR shows that 95.85% of images were classified correctly as not belonging to a class. As can be seen with the PPV, in 49.90% of cases, fabrics contained the cotton content our model categorized them as, and as the negative predicted value indicates, 95.87% of images did not belong to a class our model did not categorize them in. The F1-score tells us that the harmonic mean of precision and recall lies at 49.54%. The low standard deviation of the accuracy of the presented model shown in Table 3 indicates consistent performance across folds.

**Table 3:** Overview of multiple performance indicators applied to measure the performance of the hybrid model across five folds.

Performance indicator	Fold 1	Fold 2	Fold 3	Fold 4	Fold 5	Mean Value ± Std. dev.
RMSE	13.61%	13.07%	14.57%	13.25%	15.53%	**14.01% ± 0.92%**
MAE	6.17%	5.45%	6.92%	6.06%	7.69%	**6.46% ± 0.77%**
Accuracy	50.00%	56.15%	48.46%	49.23%	47.31%	**50.23% ± 3.09%**
Balanced Accuracy	50.00%	45.15%	48.46%	49.23%	47.31%	**50.23% ± 3.09%**
True Positive Rate	50.00%	56.15%	48.46%	49.23%	47.31%	**50.23% ± 3.09%**
True Negative Rate	95.83%	96.35%	95.71%	95.77%	95.61%	**95.85% ± 0.26%**
Positive Predictive Value	49.84%	54.58%	45.98%	49.26%	49.87%	**49.91% ± 2.75%**
Negative Predictive Value	95.84%	96.37%	95.74%	95.78%	95.60%	**95.87% ± 0.26%**
Kappa	0.4583	0.5250	0.4417	0.4500	0.4292	**0.4608 ± 0.0335**
F1-score	49.57%	54.72%	46.57%	48.69%	48.16%	**49.54% ± 2.77%**

**Table 4:** Overview of the results achieved for global RMSE, MAE, TPR, PPV, TNR, NPV, F1-score, and the 95% Wilson confidence interval (CI) for the TPR of each class.

class	RMSE	MAE	TPR	PPV	TNR	NPV	F1-score	CI
30%	13.20%	4.96%	85.00%	76.82%	97.84%	98.76%	80.50%	76.70-90.70%
40%	9.51%	3.59%	83.00%	77.70%	97.90%	98.60%	79.88%	74.50-89.10%
50%	17.09%	6.56%	82.00%	85.06%	98.78%	98.52%	83.32%	73.30-88.30%
53%	15.74%	7.98%	49.00%	45.04%	95.08%	95.72%	46.62%	39.40-58.70%
58%	11.95%	6.36%	38.00%	49.56%	96.92%	94.96%	42.80%	29.10-47.80%
60%	10.17%	4.60%	34.00%	29.98%	93.50%	94.46%	31.60%	25.50-43.70%
63%	12.52%	6.40%	13.00%	14.56%	93.34%	92.80%	13.36%	7.80-21.00%
65%	11.61%	5.33%	26.00%	28.22%	94.68%	93.88%	26.30%	18.40-35.40%
66%	10.50%	4.14%	52.00%	52.22%	95.84%	96.00%	51.58%	42.30-61.50%
80%	15.40%	9.73%	49.00%	52.00%	96.18%	95.78%	50.14%	39.40-58.70%
95%	17.50%	10.45%	52.00%	52.60%	96.08%	96.00%	51.78%	42.30-61.50%
98%	14.21%	4.95%	52.00%	47.12%	95.16%	95.98%	48.60%	42.30-61.50%
99%	19.10%	8.91%	38.00%	37.82%	94.84%	94.84%	37.62%	29.10-47.80%

Significance tests were performed on the cross-validation folds of the proposed model, comparing it to the baseline architectures DenseNet121 (33.85%, 31.92%, 44.62%, 41.15%, 33.46%, 38.85%, 38.08%, 44.23%, 33.46%, 27.69%) and Swin Transformer V2 (45.00%, 44.62%, 43.46%, 50.38%, 49.23%, 52.69%, 45.00%, 46.15%, 50.00%, 40.77%), using accuracy values. A paired t-test and a Wilcoxon signed-rank test were used with a threshold value α of 0.05 (Table 5). The paired t-test (*p* = 0.0003, highly significant) indicates a statistically significant improvement of the full proposed architecture over the DenseNet121 baseline across the cross-validation folds. The Wilcoxon signed-rank test confirms this result (*p* = 0.0020, highly significant). Similarly, the paired t-test (*p* = 0.0176, significant) indicates a statistically significant improvement over the Swin Transformer V2 baseline, which is again confirmed by the Wilcoxon signed-rank test (*p* = 0.0277, significant).

**Table 5:** Statistical comparison of the hybrid architecture against DenseNet121 and Swin Transformer V2 based on classification accuracy. Paired tests were conducted across *N* = 10 measurements (two independent runs of 5-fold cross-validation). Significance levels: * *p* < 0.05, ** *p* < 0.01, *** *p* < 0.001.

Architecture	Paired t-test	Wilcoxon signed-rank test
DenseNet121	*p* = 0.0003^***^	*p* = 0.0020^**^
Swin Transformer V2	*p* = 0.0176^*^	*p* = 0.0277^*^

**Fig 9:**
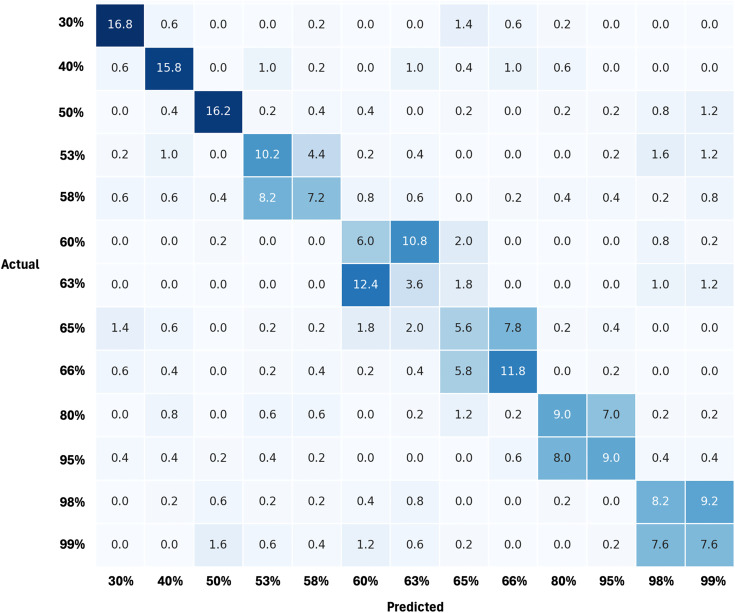
Mean confusion matrix across all five folds.

**Fig 10:**
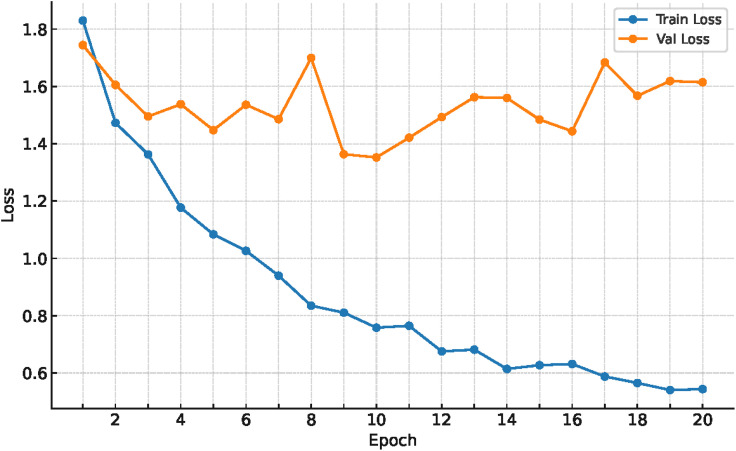
Progression curves of training and validation loss from the second run of cross-validation are shown. Training was stopped at epoch 20 with early stopping. The lowest validation loss occurred at epoch 10.

**Fig 11:**
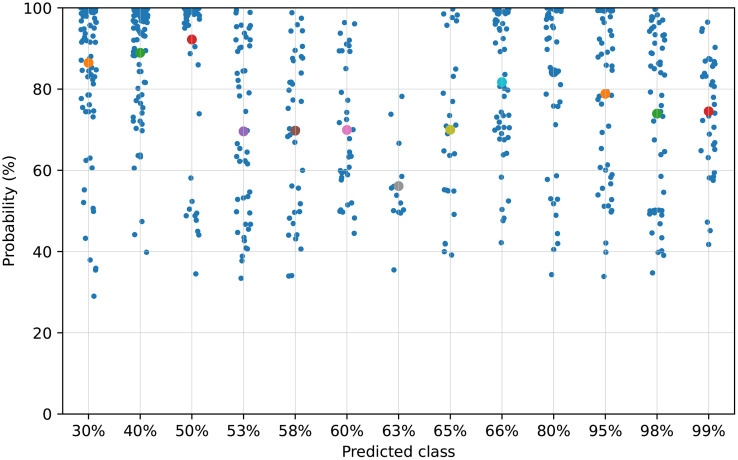
Scatter plot showing the distribution of probabilities with which the image was correctly assigned to a class. The test images (n = 653) from all 5 runs of the cross-validation are shown. The colored dots indicate the average of all individual probabilities for a class. The average probabilities per class are: class 30%: 86.47%; class 40%: 88.93%; class 50%: 92.21%; class 53%: 69.59%; class 58%: 69.76%; class 60%: 69.93%; class 63%: 56.12%; class 65%: 70.00%; class 66%: 81.71%; class 80%: 84.15%; class 95%: 78.83%; class 98%: 73.95%; class 99%: 74.53%.

Fig. 10 shows the course of the training and validation losses. Training was terminated prematurely. For the subsequent model evaluation, the weights of the epoch with the lowest validation loss, epoch 10, were used. A scatter plot showing the probabilities with which an image was correctly assigned to a class is shown in Fig 11. The mean probability of each class is above 60%, with the exception of class 63%. As shown in the confusion matrix in Fig 9, the misclassifications that do occur are mostly limited to adjacent classes, which is also reflected by the low RMSE and MAE. Additionally, there are certain error classes apparent, for example, between 53% and 58% cotton, 60% and 63% cotton, or 98% and 99% cotton. The only one that falls out of this scheme of a maximum 5% difference is the error between 80% and 95% cotton. As seen in Table 6, we conducted an ablation study to prove the effectiveness of our network and the respective innovations added. While all of our innovations did improve the overall accuracy of the architecture, the one here used combination was the best performing one, with an accuracy above 50%.

**Table 6:** Ablation study of the proposed architecture. DN = DenseNet121, Swin = Swin Transformer V2, DConv = deformable convolution layer, AFPN = adaptive feature pyramid network, 2nd FC = second fully connected layer. Paired tests were conducted across *N* = 10 measurements (two independent runs of 5-fold cross-validation). Gain denotes the accuracy difference in percentage points (pp) relative to the full architecture shown in the first row. The p-values result from paired t-tests comparing each configuration to the first-row architecture. Significance levels: * *p* < 0.05, ** *p* < 0.01, *** *p* < 0.001.

DN	Swin	DConv	AFPN	2^nd^ FC	RMSE	MAE	Accuracy	Gain	p-value
✓	✓	✓	✓	✓	**13.96% ± 1.07%**	**6.38% ± 0.77%**	**50.92% ± 3.35%**	**n/a**	**n/a**
✓	✓	✓	✓	×	15.45% ± 0.80%	7.36% ± 0.73%	48.58% ± 2.19%	−2.34	0.0288^*^
✓	✓	×	×	✓	14.25% ± 0.86%	6.75% ± 0.85%	48.12% ± 2.55%	−2.81	0.0370^*^
✓	✓	×	✓	×	14.69% ± 0.82%	7.05% ± 0.76%	47.69% ± 2.39%	−3.23	0.0047^**^
✓	✓	✓	×	×	14.52% ± 1.57%	6.97% ± 1.30%	47.35% ± 3.38%	−3.58	0.0155^*^
✓	✓	×	×	×	14.40% ± 1.34%	6.98% ± 1.10%	47.12% ± 2.80%	−3.81	0.0265^*^
✓	×	×	×	×	18.23% ± 2.33%	10.49% ± 2.45%	36.73% ± 5.28%	−14.19	0.0003^***^
×	✓	×	×	×	16.24% ± 1.35%	8.17% ± 1.12%	46.37% ± 3.50%	−4.19	0.0176^*^

## Discussion

With an RMSE of 14.01% and an MAE of 6.46%, our model shows that most predictions deviate only slightly from the true percentage values, underscoring the closeness of the estimated classes. This performance sets a new benchmark for cotton-content classification. Especially the good performance of our model for the TNR and NPV is to be emphasized. As the TNR indicates, our model classifies 95.85% of images correctly as not belonging to a class. The NPV further underscores this performance measure, showing that 95.87% of the images that were classified by our model did not belong to any other class. These measures show that our model is especially good at delimiting classes from each other. Further, our model can correctly exclude the classes whose images do not belong to it. We did achieve this with our hybrid approach, combining a modified DenseNet121 with a Swin Transformer V2. This Swin Transformer V2 enables this architecture to better extract global and local information from the data [15]. This is especially useful for detecting cotton content, as small differences in the fibers make the difference between cotton and other materials used, but the amount of those fibers makes up the cotton content of the fabric. The ablation study shown in Table 6 shows the influence of individual components on accuracy, RMSE, and MAE. Densenet121 baseline achieves the lowest accuracy and also the highest RMSE and MAE. Swin Transformer V2, on the other hand, already achieves higher values with 46.37% accuracy. The combination of both baseline models increases accuracy to 47.12% while reducing the RMSE and MAE values. Based on the close results, it can be assumed that the Swin Transformer contributes a higher proportion to the classification. The impact of each modification to the architecture is quantified in Table 6. If the second fully connected layer is removed, accuracy decreases by 2.34 pp (*p* < 0.05). If AFPN or the DConv layer is removed, there are statistically significant reductions in accuracy of 3.23 and 3.58 pp, respectively. The sharpest decline occurs when the hybrid model is dissolved and individual models are used (up to 14.19 pp). These results show that the performance of the architecture arises precisely from the specific combination of the individual architectural components.

To assess the statistical significance of the results obtained across the cross-validation folds, the performance of the hybrid architecture was compared with the baseline models DenseNet121 and Swin Transformer V2 using two statistical significance tests, the paired t-test and the Wilcoxon signed-rank test based on accuracy. The p-values obtained in comparison to DenseNet121 ($p_{t-test}$=0.0003, $p_{wilcox}$=0.0020) are below the significance level of 0.05. This finding suggests that the observed enhancement in performance of the hybrid architecture is not merely random, but rather, it occurs in a consistent manner across the individual folds. The integration of local (Densenet121) and global (Swin Transformer V2) feature extraction demonstrates a notable enhancement in performance. In comparison to Swin Transformer V2, significant differences are also observed ($p_{t-test}$ = 0.0176, $p_{wilcox}$ = 0.0277). These results indicate that the hybrid architecture consistently outperforms both baselines, rather than performing merely comparably. The ablation study in Table 6 still suggests a stronger contribution of the Transformer component to the overall performance. The Confusion Matrix in Fig 9 shows that the misclassifications our model does have, most commonly lie in neighboring classes. This qualitative observation is supported by the quantitative error metric MAE. Specifically, the average MAE across all five cross-validation folds is 6.46%. The average difference between cotton grades is 5.75%. The average MAE achieved thus shows that the predicted cotton content deviates on average by approximately one neighboring class interval from the actual class. Table 4 shows a more detailed representation of the MAE values achieved per class.

This behavior can be illustrated using the 63% cotton-content class as an example. For this class, an MAE of 6.40% was obtained. The neighboring classes correspond to cotton contents of 58% (−5% distance from 63% class), 60% (−3% distance from 63% class), 65% (+2% distance from 63% class), and 66% (+3% distance from 63% class). Thus, the value of the MAE largely corresponds to the distance to these neighboring classes, confirming that most classification errors are limited to nearby categories and not to distant ones. This pattern is also reflected in the confusion matrix, where the most frequent misclassifications for the 63% class occur in the 60% and 66% categories, both of which lie within the MAE interval. The MAE values for many classes lie in a narrow range between approximately 4–7% (30%: 4.96%, 40%: 3.59%, 50%: 6.56%, 60%: 4.60%, 65%: 5.33%, and 66%: 4.14%). These sizes correspond to deviations from the nearest classes, particularly in densely sampled regions of the class space. In combination with the confusion matrix (Fig 9), this indicates that most misclassifications are local rather than distant. For the 80% cotton class, the MAE of 9.73% is consistent with the large numerical gap to the neighboring classes at 66% and 95%, meaning that even misclassifications to adjacent classes lead to comparatively large absolute errors. For the 95% class, the MAE of 10.45% can be explained by the asymmetric spacing to its neighboring classes, with a large distance to 80% and a much smaller distance to 98%. For the 98% class, the MAE of 4.95% is consistent with its close proximity to both neighboring classes, 95% and 99%, which are separated by 3 and 1 percentage points (pp), respectively. The higher MAE of 8.91% for the 99% class reflects the influence of occasional misclassifications to more distant classes, while the confusion matrix shows that most errors occur in the adjacent 98% category. This is a known problem that networks struggle with classification problems as the number of classes increases because algorithms work less good, when applied to multiple decision boundaries simultaneously [34]. CNNs are known to struggle, especially for classifying data which has inner-class variability and outer-class similarity [35]. We do have this problem in our domain, as the fabric images in the classes vary in color, weaving, and structure, but the different classes are only differentiated by their cotton content, making it nearly impossible to distinguish by the human eye. These misclassification clusters are especially interesting as there are clusters visible, where the misclassifications are more common. While misclassifications are rare in the categories of 50% cotton and less, the clusters tend to form in the same 10% batches rather than beyond those borders. For the classes 50% and below, the small number of errors is explainable with the comparatively high 10% difference of these classes. In these classes, the highest accuracy is achieved with 85.00%, 83.00%, and 82.00% (for the classes 30%, 40%, and 50% respectively). In some cases, for example between 65% and 66% or 98% and 99%, the model tends to wrongly classify the classes with cotton contents rather close to each other, which is logical as the differences between those are lower as well. An exception to this are the classes of 80% and 95% cotton, which get misclassified commonly, despite a 15% difference. Examining the performance of the individual classes reveals that low TPRs are achieved for classes 60% to 65% (34%, 13%, and 26%), indicating that correctly classifying this cotton content is more difficult and less reliable. This is followed by the 58% and 99% classes, each of which achieves a TPR of 38.00%. Other exceptions which seem counterintuitive are 53% and 58%, where both neighboring classes, 50% and 60%, are closer than the ones in the misclassification cluster. The same is true for the 60% and 63% clusters. A potential explanation for all of these counterintuitive clusters is the neighboring ones. As the 98% and 99% as well as the 65% and 66% clusters have only one percent difference, the model might be able to differentiate images that do not belong in these classes, but then only learn that they are not either of them, leading to mistakes in the classes in between. This might occur with the cluster between 60% and 63% as well, as it neighbors the 65% and 66% cluster. The 53% and 58% clusters are not as explainable, being nearly not misclassified with the 50% and 60% classes, which do not have closer neighbors. Since these results are based on purely visual data, the classification must be based on information in the RGB images. Since less information about the information content of the cotton content is encoded here, there is an accuracy ceiling compared to spectroscopy-based methods.

Despite the good results achieved, potential biases in the dataset that may affect the generalization of the model should be taken into account. The images of the dataset were acquired under specific and homogeneous conditions, including consistent indoor lighting, fixed camera positioning, and uniform backgrounds. While this setup ensures high image quality and reduces noise during training, it may limit the model’s robustness when deployed in real-world recycling or inspection scenarios. In such environments, fluctuations in lighting, shadows, camera angles, surface contamination, and background textures can occur and can lead to a deterioration in performance if the model is not adapted to these conditions. Since the fabric samples were collected in Bangladesh, the dataset has a geographical and industrial context. As a result, the visual characteristics and structural properties of the textiles primarily reflect manufacturing practices, raw materials, machinery, and environmental conditions typical of this region. Fabrics produced in other geographic locations may differ in fiber composition, yarn processing, weaving or knitting techniques, and finishing procedures. These differences could affect the learned feature representations and, consequently, reduce the model’s predictive performance when applied to textiles from other regions or production chains. This dataset contains only non-personal images of fabric samples and poses no privacy risks. Furthermore, it should be noted that the dataset did not contain any explicit metadata about the source, such as the manufacturer or production batch. Although the StratifiedKFold method ensured disjoint divisions, it cannot be completely ruled out that sources overlapped, which may affect the results obtained. Finally, the accuracy and error margin of the thread-counting machine used to generate the labels are not explicitly documented. Any systematic or random measurement errors introduced at this stage directly propagate into the training and evaluation metrics. Such label uncertainty may limit the model’s achievable performance and generalization capability.

### Contextualization of the results

To contextualize the achieved RMSE of 14.01%, the proposed hybrid architecture is compared with the baseline models. DenseNet121 yields an RMSE of 17.87% (+3.86 pp), while the Swin Transformer also exhibits a higher RMSE (+2.34 pp), noting that lower RMSE values indicate better performance. The values in parentheses indicate the deviation in percentage points from the hybrid architecture.

In [7], the fiber content of blended fabrics is predicted using NIR and CNNs. The study aims to improve the accuracy and robustness of composition analysis. Several methods are evaluated, showing a considerable variation in RMSE values for cotton. Random Forest achieves 17.70%, an encoder-based model 18.20%, and the ResNet model reaches an RMSE of 13.70%, the best model reaches an RMSE of 11.70%. In [10], Paz and Sousa investigate the ability of NIR and MIR to determine the cotton content in blended fabrics. Using the NIR and MIR method, an RMSE of 7.8% and 8% was achieved on textiles with cotton, polyester and other material. For textiles with cotton and polyester RMSE of 3.6% (NIR) and 6.5% (MIR) are achieved. Further studies that also used NIR, such as Xia et al. [8] and [11], achieved RMSE values of 0.65% and 2.10%, respectively. However, a direct comparison between the studies that used NIR and our study is hardly possible, as the underlying methodology differs significantly. While spectroscopy captures information that is directly linked to the cotton content via wavelength absorption, RGB imaging is based solely on the visual appearance of the fabrics. These measurement methods therefore encode less information than spectroscopy-based methods and are thus intrinsically limited. Even if a perfectly functioning model were available, it would not be able to distinguish between fabrics with different cotton content but identical visual appearance. This means that there is an inherent accuracy ceiling for these visual methods, and the RMSE of NIR approaches should therefore be understood as a measurement-driven accuracy ceiling rather than competition for visual detection methods.

To our knowledge, the work by Islam et al. [14] represents the only study that adopts a visual approach to classify cotton content in fabrics and therefore provides a comparison to our method. With the VGG16 model and transfer learning, they achieve an RMSE of 7.56%. However, this study addresses a different experimental setting and the RMSE value is therefore not directly comparable to our setting. Although our RMSE of 14.01% did not surpass their RMSE of 7.56%, this does not indicate lower performance but results from a more constrained and real-world oriented problem formulation. First, we employed a peer-reviewed dataset with an equal number of images across all categories [4]. In addition, our dataset contains RGB images captured with a smartphone camera. Furthermore, we use a stratified 5-fold cross-validation to prevent lucky splits in our training and test sets. Without cross-validation, there is no guarantee that a lucky split will not occur, which makes the results less reliable [36]. In contrast to our approach, Islam et al. [14] use a photometric stereo sensor, where four color images are captured for each fabric sample under different illumination conditions [14,37]. Such data acquisition simplifies the prediction task compared to classification from single RGB images. This introduces additional challenges in ensuring that images of the same fabric are strictly separated between the training and testing sets. The absence of cross-validation further increases this probability, as different data splits may include varying numbers of the same images in the training and testing sets. Therefore, we establish a reproducible cross-validated camera-based benchmark using stratified 5-fold cross-validation.

### Use-case implications

As we set a new benchmark for visually classifying the cotton content of fabrics, we show that a hybrid approach is applicable for sufficient classification. By not depending on NIR spectrography or other complicated techniques, our approach can be less costly and complex. The usability of only this approach for tasks such as correctly labeling fabrics is, even with this new benchmark, limited, but it could be used to pre-classify the fabrics to speed up other processes. Here, after the fabrics are delivered, our system could be applied for an initial sorting task. The same goes for the application in recycling tasks, where a lot of different fabrics have to be sorted. In this area, it is not essential to classify correctly on the percent, but DL-methods like ours can help to organize the fabrics in similar groups.

### Circular economy implications

We set out to help solve issues of the circular economy, as well as applications in the textile industry, and find possible applications for recycling practices. These are important for our world’s sustainable development, as recycling and waste reduction through reuse are key targets for the SDG goal 12, sustainable consumption and production patterns [2]. A possible route for the recycling industry to enhance its efficiency, especially through the application in sorting tasks are benefits of machine-learning approaches [38]. Furthermore, automating the sorting process is a strong hope to solve a key challenge of the industry, as it improves the economic viability of textile waste recycling [39]. As this sorting task could be a key use case of our architecture, we propose a possible approach to enhance the efficiency and economic viability of textile recycling, which helps to come a little closer to reaching the UN SDGs.

## Conclusion

With the proposed hybrid architecture approach, we set a new benchmark for visual approaches to classifying fabrics based on their cotton content with state-of-the-art validation techniques. Achieving an average RMSE of 14.01% we show that the combination of DenseNet121 and Swin Transformer V2 is suited well for the task of classification of cotton content in fabrics. We further prove the effectiveness of modifying DenseNet121 with multiple innovations. AFPN make the network consider features extracted at different levels and enhance the performance of our architecture, as well as an added DConv layer, which enhances the network’s capability of focusing on specific structures in the images. The additional use of a second FC layer after the concat fusion further enhanced the classification capacity of our network. Our proposed architecture is an example of how DL methods could be applied in fabric classification, for example, for pre-sorting use cases in recycling or other application fields. As outlined before, automation of the sorting process is a key challenge in the recycling industry. Architectures like ours could be applied at an early stage of the process, for example, when recyclable products are delivered at a processing facility, to initially sort the products into categories after their cotton content. This would potentially streamline the recycling process by reducing time and labor intensity.

### Limitations

We set a new benchmark for visual fabric classification; however, our work does come with some limitations. Our model does have a low RMSE and MAE; however, it tends to incorrectly classify neighboring classes. While we achieve good performance metrics, the performance of our model is not enough for high precision tasks, such as determining the cotton content of a fabric to the percent. This must be viewed against the background of the inherent limitations in accuracy that visual methods face compared to spectrographic methods. Visual methods are used because they are quick and easily scalable; however, they measure a proxy for the cotton content as they capture the visual appearance. It is therefore expected that lower performance will be achieved compared to, e.g., spectroscopy, where the chemical composition is directly part of the measurement signal. Since it is conceivable for visual images that identical cotton contents lead to identical visual appearance, zero-error performance is impossible due to this accuracy ceiling of visual methods alone. Thus, a 14.01% RMSE can be seen as a strong achievement within the visual domain, given its practical advantages; however, it offers inherent limitations in achieving the highest performance. Another limitation arises from the fact that we classified based on cotton content. Additional modifications in this vein could also improve the relevance to practical tasks, as fabrics like wool or polyester are common as well. Furthermore, the classes utilized for our model classification came from our dataset. Here, a different division of the classes, for example, dividing them into 5% increments, could be implemented.

### Future work

Future research could focus on further analyzing the here occurred misclassification clusters. While the problems of CNNs with multiclass problems and common workarounds are extensively researched, this phenomenon is not. Additionally, the proposed architecture could be applied in different areas for similar tasks to further explore the potential of a DenseNet-Swin combination. Similarly, our modifications to DenseNet121 could be applied in different areas to research possible performance enhancements. To find reasons for the misclassification cluster that occurred in our work, explainable AI tools could be applied. Here, common tools like LIME and SHAP proved performance across different application fields [40]. Additionally, future work could explore the application of preprocessing filters like the Gaussian filter, which has been proven to enhance accuracy in multiple application fields [41–43]. Following this example, Gaussian filtering could be tested in our use case as well. Our approach could further be applied to other fields of research to explore the possible performance gains when applying hybrid architectures with the Swin Transformer V2. For lowering costs and enhancing precision in other application fields of the textile industry, the classification of different textures and weaving types could be an interesting approach.
